# The illusory-truth effect and its absence under accuracy-focused processing are robust across contexts of low and high advertising exposure

**DOI:** 10.1186/s41235-025-00628-3

**Published:** 2025-05-13

**Authors:** Raoul Bell, Lena Nadarevic, Laura Mieth, Axel Buchner

**Affiliations:** 1 Department of Experimental Psychology, Faculty of Mathematics and Natural Sciences, Heinrich Heine University Düsseldorf, 40225 Düsseldorf, Germany; 2 Department of Psychology, School of Social Sciences, University of Mannheim, 68161 Mannheim, Germany; 3 Department of Psychology, Charlotte Fresenius Hochschule, 65185 Wiesbaden, Germany

**Keywords:** Illusory-truth effect, Advertising exposure, Context effect, Fluency, Truth judgments

## Abstract

In present-day digital environments, people frequently encounter content from sources of questionable trustworthiness. Advertising is an untrustworthy source because its purpose is to persuade consumers rather than to provide impartial information. One factor known to enhance the perceived truth of advertising claims is repetition: Repeated advertising claims receive higher truth ratings than novel advertising claims. The phenomenon that repetition enhances processing fluency which enhances truth judgments is known as the illusory-truth effect. Does repetition always enhance truth judgments? For instance, does repetition enhance truth judgments even in contexts with extensive advertising exposure in which enhanced processing fluency could be used to classify a statement as likely coming from an untrustworthy source? In two experiments, we examined the illusory-truth effect by presenting participants with product statements in an exposure phase and collecting truth judgments for both repeated and new statements in a test phase. In a low-advertising-exposure condition, most of the statements were labeled as scientific studies while in the high-advertising-exposure condition, most of the statements were labeled as advertising. When participants read the product statements in the exposure phase, a typical illusory-truth effect was obtained: In the test phase, repeated statements received higher truth ratings than new statements. However, when participants were instructed to adopt an accuracy focus at encoding by judging the truth of the product statements, new statements were judged to be as true as repeated statements. Both the illusory-truth effect and its absence under accuracy-focus instructions were found to be robust across different levels of advertising exposure.

## Significance

The ability to distinguish truth from falsity is fundamental for informed decision-making. However, in present-day digital environments, this task has become quite challenging. Due to the easy and effective dissemination of information in the Internet age, individuals frequently encounter content from sources of questionable trustworthiness (Kozyreva et al., 2020). An example for such an untrustworthy source is advertising (Bell et al., 2022) because its purpose is to persuade consumers rather than to provide impartial information (Boush et al., 2009; Friestad & Wright, 1994). A high proportion of information from advertising in online contexts poses a significant challenge for judging the truth of information: The strategies people traditionally rely on to determine the truth of information in offline contexts may no longer be effective (Campbell & Grimm, 2019). It is, therefore, critical to understand how exposure to untrustworthy sources influences truth judgments. Here, we examined how the perceived truth of product statements is influenced by advertising exposure. Participants viewed statements under high- or low-advertising-exposure conditions (60% or 20% advertising). Later, repeated statements were judged to be more truthful than new statements, consistent with the illusory-truth effect. However, when participants adopted an accuracy focus during exposure, the illusory-truth effect disappeared: Repeated and new statements were judged to be equally truthful. Crucially, variations in advertising exposure did not influence truth judgments, highlighting the robustness of the illusory-truth effect and its modulation by accuracy focus. These findings suggest that people fail to adjust their truth judgments to differing levels of advertising exposure.

## Introduction

Due to the abundance of information encountered everyday, it is not feasible to determine the truth of every piece of information through direct observation or logical deduction. As a result, people rely on simple heuristics to judge whether a piece of information is true. Specifically, people often evaluate the truth of information based on how easy it is to process the information. Information that is fluently processed because it aligns closely with existing knowledge or beliefs is typically accepted more readily as true than information that is less fluently processed (Unkelbach & Rom, 2017). Prior exposure to a statement also increases how fluently the statement is processed and, as a consequence, how likely it is judged to be true independent of whether the statement is actually true (Hasher et al., 1977). This phenomenon has been called the *illusory-truth effect* (Dechêne et al., 2010).

A typical paradigm for examining the illusory-truth effect is to present participants with a list of statements in an exposure phase (Nadarevic, 2022; Udry & Barber, 2024). In a subsequent test phase, participants judge the truth of the statements on a scale ranging from “definitely false” to “definitely true.” Some of the test-phase statements are repeated from the exposure phase and others are new. Provided that participants do not process the information with a focus on accuracy in the exposure phase (more on this boundary condition below), an illusory-truth effect emerges even after a short interval between the exposure phase and the test phase: Truth ratings are higher for repeated statements than for new statements (Nadarevic & Erdfelder, 2014).

When we interact with relatives, friends, and colleagues, we generally trust most of the information they share with us to be truthful, an expectation that has been referred to as one of the “tacit assumptions underlying the conduct of conversations in daily life” (Skurnik et al., 2005). In such contexts, it may well be a useful heuristic to rely on cues of prior exposure such as processing fluency to determine the truth of a statement. However, this heuristic can be exploited to manipulate truth judgments in the service of commercial or political agendas. For instance, advertising statements may be repeated to increase their processing fluency, making them more likely to be judged as true by consumers. When used excessively, this strategy may backfire. Specifically, if the same advertising message is repeated over and over again, the illusory-truth effect may eventually reverse as people start perceiving the repetition of the message as a persuasive attempt (Koch & Zerback, 2013). Here, we built on this research by examining the effects of advertising exposure on the illusory-truth effect more broadly. Specifically, we tested whether high levels of exposure to different advertising messages, typical of many modern digital environments, change the way people use cues of prior exposure in their truth judgments. When consumers are exposed to excessive amounts of advertising, the ecological correlation between prior exposure and truth may be called into doubt. This leads to the question of whether the relative proportion of statements from trustworthy and advertising sources changes how people react to cues of prior exposure when making judgments of truth.

The answer to this question is not straightforward, as different lines of research point to different answers. On the one hand, the illusory-truth effect has been observed with various stimulus materials, ranging from trivia statements such as “Lithium is the lightest of all metals” (Hasher et al., 1977), health-related statements such as “Antibiotics do not help against SARS-CoV-2” (Unkelbach & Speckmann, 2021), news headlines such as “AP poll: 62% disapprove of how Trump’s handling his job” (Calvillo & Smelter, 2020; Pennycook et al., 2018), meaningless statements such as “A ma is bigger than an omp” (Unkelbach & Rom, 2017), and advertisements such as “Billabong shampoo leaves hair shiny with no residue” (Hawkins & Hoch, 1992; Law & Hawkins, 1997; Roggeveen & Johar, 2002). The illusory-truth effect also occurs for information from untrustworthy sources (Begg et al., 1992; Henkel & Mattson, 2011; Nadarevic et al., 2020). Furthermore, warnings such as “disputed by 3rd party fact checkers” do not fully eliminate the illusory-truth effect (Pennycook et al., 2018; see also Nadarevic & Aßfalg, 2017). These results suggest that the illusory-truth effect generalizes readily across different stimulus materials and contexts.

On the other hand, there is evidence that the effect of prior exposure on truth ratings is context-dependent. Specifically, warning participants that a relatively large proportion of the statements in the exposure phase had been false has been reported to substantially decrease the illusory-truth effect, supporting the assumption that people rely less on cues of prior exposure to infer truth if it is expected that less than the bulk of the communicated information is truthful (Jalbert et al., 2020). Furthermore, Unkelbach and Stahl (2009) have shown that, when instructing participants that *all statements from the exposure phase are false*, the interpretation of processing fluency changes in line with these instructions. These results are in line with a broader model of bounded rationality according to which people may learn to interpret processing fluency in a context-dependent way: If people have reasons to believe that false statements are more fluently processed in a given context, processing fluency may be used as a cue for falsity rather than as a cue for truth (Unkelbach, 2007; see also Corneille et al., 2020; Scholl et al., 2014; Skurnik et al., 2000). However, real-world contexts are more ambiguous and less straightforward than scenarios suggesting that *all* of the statements are false. Specifically, it is more likely that everyday contexts may contain varying proportions of true or false information than that they contain only true information or none at all. Furthermore, everyday contexts may include untrustworthy sources such as advertising, implying that the information from the source does not have to be blatantly false but there is still valid reason to doubt its veracity (Boush et al., 2009; Friestad & Wright, 1994). To understand how people judge truth in situations where they are exposed to varying amounts of information from untrustworthy sources, we examined the impact of contexts with high or low advertising exposure on the illusory-truth effect.

In the present experiments, participants read a series of statements about products. Two conditions were contrasted: In a low-advertising-exposure condition, 60% of the product statements referred to scientific studies, 20% were advertising statements, and 20% were presented without a source. In a high-advertising-exposure condition, 60% of the product statements were advertising statements, 20% referred to scientific studies, and 20% were presented without a source. This approach builds on prior evidence that these base rates of trustworthy and untrustworthy information are processed, represented, and remembered, as they influence memory judgments (Bell et al., 2022). Specifically, when asked to retrieve the source from memory, participants in the high-advertising-exposure condition were much more likely to guess that a statement originated from advertising than participants in the low-advertising-exposure condition, particularly if the statement was recognized as having been present in the exposure phase. While this evidence strongly suggests that differences in the proportion of advertising and scientific statements are cognitively represented and can influence memory judgments, it remains unclear whether this contextual knowledge is taken into account when judging the truth of information.

In Experiment 1, we tested whether the illusory-truth effect is robust across contexts with high and low advertising exposure. Participants were required to read the product statements in the exposure phase, thereby engaging with the statements in a non-evaluative manner. Under these conditions, we expected the repeated statements to be associated with higher test-phase truth ratings than the novel statements in the low-advertising-exposure condition. However, it is unclear whether the illusory-truth effect generalizes to the high-advertising-exposure condition. If high advertising exposure prompts participants to interpret cues of prior exposure as being no longer indicative of truth or even as being indicative of falsity, the illusory-truth effect may be abolished or even reversed in the high-advertising-exposure condition.

In Experiment 2, participants were required to judge the truth of the statements in the exposure phase. Previous research has shown that the illusory-truth effect is abolished when participants’ attention at exposure is directed toward the accuracy of information and the to-be-judged information is equally likely to be true or false (Brashier et al., 2020; Calvillo & Smelter, 2020; Nadarevic & Erdfelder, 2014). Here, we examine whether focusing on accuracy prompts participants to form an episodic representation of the proportion of truthful or untruthful information in the exposure phase. If the information mainly originates from a trustworthy source, the focus on accuracy may reinforce the validity of relying on cues of prior exposure to infer truth, thereby strengthening the illusory-truth effect. However, when advertising exposure is high, a focus on accuracy may lead participants to critically evaluate the statements they are exposed to, potentially even shifting the interpretation of cues of prior exposure from being indicative of truth to being indicative of falsity. As a result, repeated statements should receive lower truth ratings than new statements in the high-advertising-exposure condition.

## Experiment 1

### Methods

#### Participants

To obtain the large sample sizes necessary to detect even a subtle interaction effect between statement type (new, advertising, no source, or study) and advertising exposure (low or high) if it existed, participants were recruited via the online-access-panel provider *Cint* (https://www.cint.com/). We aimed at collecting valid data of about 500 participants and ended data collection the day this criterion was reached. The data of 100 participants who had started the exposure phase had to be excluded because these participants did not complete the experiment or withdrew their consent into the use of their data. The final dataset comprised the data of *N* = 527 participants (229 females, 297 males, and 1 diverse) with a mean age of 46 (*SD* = 15) years, most of whom (500) were German native speakers. The sample was characterized by a diverse range of education. Participants were randomly assigned to either the low-advertising-exposure group (*n* = 263) or the high-advertising-exposure group (*n* = 264). A sensitivity analysis with G*Power (Faul et al., 2007) showed that, with a sample size of *N* = 527 participants and a multivariate approach to the repeated-measures analysis (O'Brien & Kaiser, 1985), an interaction between statement type and advertising exposure on the truth ratings as small as η_p_^2^ = 0.03 could be detected at an α level of 0.05 with a statistical power of 1–β = 0.95.

#### Materials

For the study, 240 statements about products were created, such as “*The nutrients in Blofensir's product contribute, among other things, to the normal functioning of the nervous system, support energy metabolism, protect cells from oxidative stress and help reduce tiredness and fatigue*.” Each product statement comprised a different brand name that had been created with the pseudoword generator *wuggy* (Keuleers & Brysbaert, 2010). Examples are *Yabu, Ibutu, Yorido, Pulki, Suzzy*, and *Sentis*. The statements were selected based on an online norming study in which *N* = 61 participants judged the product statements. Participants in the norming study were informed that they would see a series of statements. They were told that some of these statements were advertising whereas other statements summarized the results of scientific studies in which comparative product tests had been carried out according to strict scientific criteria. Each statement was presented for 5 s before the rating scale was displayed. Participants were asked: “Does this statement originate from advertising or a study?” Participants had to rate the statement on a Likert-type scale ranging from − 3 (definitely advertising) to + 3 (definitely study). Each statement remained on the screen until participants confirmed their judgment by clicking on a “continue” button. From the statements included in the norming study, 120 statements were selected that were judged to be on average equally likely to originate from advertising or a scientific study (*M* = 0.00, *SD* = 0.29, min =  − 0.52, max = 0.54).

#### Procedure

The online experiment was implemented using SoSci Survey (Leiner, 2019) and made available through https://www.soscisurvey.de. Participation was only possible with a desktop or laptop computer. Before starting the study, participants were asked to participate alone in a distraction-free and quiet environment.

In the instructions for the *exposure phase*, participants were informed that they were about to read a series of statements about products. They were further informed that some of these product statements were advertising whereas other statements summarized the results of studies grounded in science in which comparative product tests had been carried out using strict scientific criteria. Participants were made aware that a label would indicate which of the product statements were advertising and which summarized the results of scientific studies. Participants were also informed that for some statements, no source would be given. Each participant read a subset of 60 product statements, randomly selected for each participant from the set of the 120 product statements selected for the experiment. In the low-advertising-exposure group, participants saw 12 advertising statements (20%), 12 statements for which no source was given (20%), and 36 study statements (60%). In the high-advertising-exposure group, participants saw 36 advertising statements (60%), 12 statements for which no source was given (20%), and 12 study statements (20%).

**Fig. 1:**
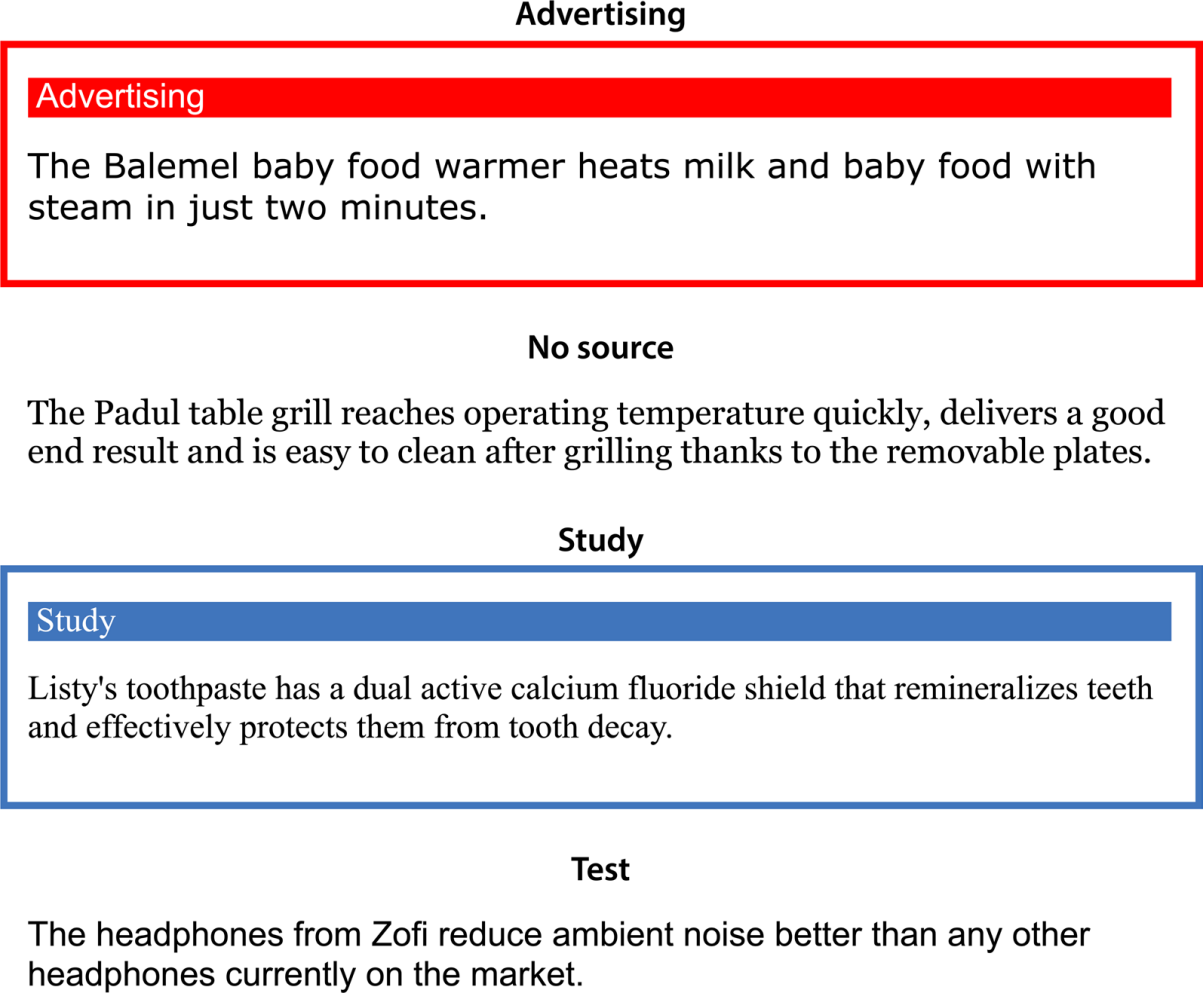
Examples of the (advertising, no source, and study) exposure-phase and test-phase statements used in the present experiments

Figure 1 displays examples of the designs of the different statements. Statements types were presented with salient labels to help participants differentiate among advertising statements, study statements, and statements without a source, consistent with previous research on the effects of advertising exposure on source monitoring (Bell et al., 2022). To further support discrimination and source monitoring, the statements types were additionally distinguished by distinct fonts. An advertising statement was labeled as “Advertising” in 24 pt white Arial font against a bright red background. The statement itself was presented in 24 pt black Verdana font. The label and the statement were enclosed by a 5 pt solid red frame. A statement for which no source was given was displayed in 24 pt black Georgia font against a white background without label or colored frame. A study statement was labeled as “Study” in 24 pt white Times New Roman font against a dark blue background. The study statement was presented in 24 pt black Times New Roman font. Both the study statement and the study label were enclosed in a 5 pt solid dark blue frame.

The statements were presented one after another in a new random order for every participant. Each statement was presented for 5 s until a “Continue” button appeared. The statement remained on the screen until participants clicked the “Continue” button, thereby starting the next trial.

When all of the statements had been presented, a distractor task followed in which participants had to solve ten simple arithmetic problems such as “16 − 9 = ” by typing the correct solution into a text field. The median duration of the distractor task was about 50 s.

At the beginning of the *test phase*, participants were informed that they would once again see a series of statements about products and that their task was to rate each statement for its veracity. Participants saw 120 statements: 60 statements from the exposure phase, randomly intermixed with 60 new statements participants had not seen before. Each participant saw the statements in a different random order. Each statement was presented in 24 pt black Arial font against a white background. For each statement, participants were asked: “Do you think this statement is false or true?” The statement had to be judged on a 6-point Likert-type truth rating scale (*1* = *definitely false, 2* = *false, 3* = *more likely false, 4* = *more likely true, 5* = *true, and 6* = *definitely true*).

At the end of the experiment, participants were informed that all brands were fictional. They were given the opportunity to withdraw their consent to the use of their data (given at the start of the experiment). Then, they were debriefed and thanked for their participation.

### Results

**Fig. 2:**
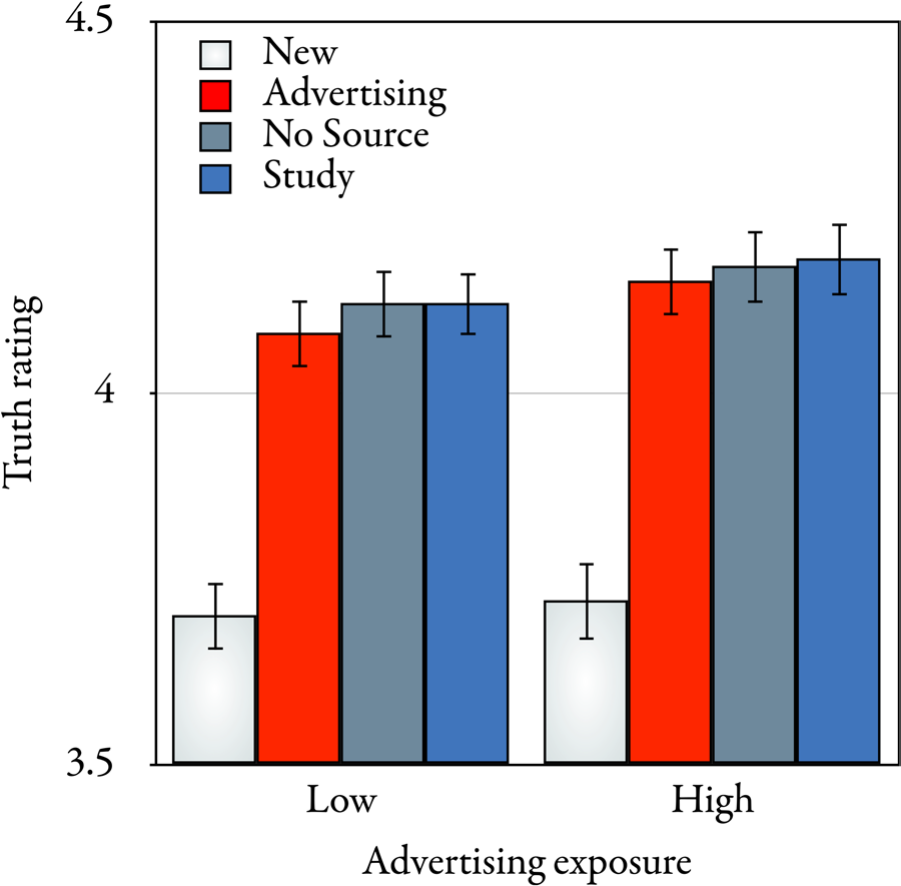
Mean test-phase truth ratings on a scale ranging from 1 (*definitely false*) to 6 *(definitely true*) as a function of (new, advertising, no source, or study) statement type and (low or high) advertising exposure in the test phase of Experiment 1. The error bars represent the standard errors of the means

The mean test-phase truth ratings are displayed in Fig. 2. A multivariate approach was used for all repeated-measures comparisons (O'Brien & Kaiser, 1985). In the present application, all multivariate test criteria correspond to the same exact *F*-statistic that is reported. The α level was set to 0.05 for all comparisons reported here. A 4 × 2 repeated-measures analysis of variance with statement type (new, advertising, no source, and study) as a repeated-measures factor, advertising exposure (high and low) as a group factor, and test-phase truth ratings as the dependent variable revealed that statement type had a significant effect on the test-phase truth ratings, *F*(3,523) = 50.90, *p* < 0.001, η_p_^2^ = 0.23. Helmert contrasts were used to analyze the differences in the test-phase truth ratings across the different statement types. New statements were rated as being less true than the repeated statements, *F*(1,525) = 152.64, *p* < 0.001, η_p_^2^ = 0.23, which is evidence of an illusory-truth effect. Advertising statements did not lead to significantly lower test-phase truth ratings than the other repeated statement types, *F*(1,525) = 3.80, *p* = 0.052, η_p_^2^ = 0.01. Test-phase truth ratings did not differ significantly between statements for which no source was displayed and study statements, *F*(1,525) = 0.03, *p* = 0.865, η_p_^2^ < 0.01. In summary, an illusory-truth effect was obtained in Experiment 1, but source did not significantly influence the test-phase truth ratings.

Advertising exposure had no significant main effect on the test-phase truth ratings, *F*(1,525) = 0.81, *p* = 0.367, η_p_^2 ^< 0.01. There was no significant interaction between advertising exposure and statement type, *F*(3,523) = 0.31, *p* = 0.815, η_p_^2 ^< 0.01. Specifically, the interaction between advertising exposure and the contrast between new and repeated statements was not significant, *F*(1,525) = 0.32, *p* = 0.575, η_p_^2 ^< 0.01.

### Discussion

Evidence of an illusory-truth effect was observed in Experiment 1. Repeated statements consistently received higher test-phase truth ratings than new statements, irrespective of whether or not the statements were labeled as advertising or scientific studies in the exposure phase. This illusory-truth effect was robustly observed across conditions of low and high advertising exposure. The interaction between advertising exposure and the contrast between new and repeated statements was not significant, even though the large sample size provided considerable sensitivity to detect such an effect if it existed. The sample effect size associated with this interaction was negligible (η_p_^2 ^< 0.01), indicating that advertising exposure had no meaningful influence on the contrast between new and repeated statements. These findings indicate that high advertising exposure does not abolish or reverse the illusory-truth effect, implying that people continue to rely on cues of prior exposure to infer the truth of statements even in such situations of high epistemic uncertainty.

As a side note, it seems pertinent to mention that, while prior exposure had a significant effect, the source labels paired with the statements during the exposure phase had no significant effect on the test-phase truth ratings. Descriptively, advertising statements received lower test-phase truth ratings compared to statements without a source and study statements. However, this difference did not reach significance, despite the large sample size, and the sample effect size associated with the contrast between advertising statements and the other statement types was negligible (η_p_^2^ = 0.01). This suggests that advertising-disclosure warnings may do little, if anything, to protect consumers from the persuasive effect of repeated exposure, consistent with previous analyses highlighting the limited efficacy of warnings on altering consumer judgments (Bell et al., 2021; Pennycook et al., 2018).

## Experiment 2

A potential limitation of Experiment 1 is that participants were instructed to read the statements in the exposure phase. These instructions may have caused a comparatively uncritical engagement with the exposure-phase statements. Even though such an uncritical stance may be representative of how people spontaneously engage with information in many everyday contexts (Nadarevic, 2022), the question arises as to whether advertising exposure may have more influence in contexts in which people evaluate the presented information more critically, in line with the previous studies demonstrating that a focus on accuracy fundamentally changes how people process false and untrustworthy information (e.g., Capraro & Celadin, 2023; Pennycook et al., 2020, 2021). Experiment 2 served to test whether the base rate of trustworthy information would modulate the illusory-truth effect when participants were required to evaluate the truth or falsity of the statements in the exposure phase.

Previous research (Brashier et al., 2020; Calvillo & Smelter, 2020; Nadarevic & Erdfelder, 2014) has shown that the illusory-truth effect is absent when participants are required to focus on accuracy during the exposure phase. One explanation for this finding is that the requirement to provide truth ratings during the exposure phase may lead people to evaluate the statements for accuracy and, in doing so, encode “true” or “false” tags with the statements. If these “true” and “false” tags can later be retrieved when the statements are repeated, people may rely on the retrieved evaluations rather than on fluency to judge the truth of the statements. If this truth-tagging hypothesis is correct, then the illusory-truth effect should be absent in Experiment 2, and those statements that have received lower truth ratings in the exposure phase—for instance, advertising statements—should also receive lower truth ratings in the test phase, irrespective of the level of advertising exposure.

Another explanation for the absence of the illusory-truth effect when the focus is on accuracy is based on the assumption that an accuracy focus leads participants to develop a broader representation of the overall likelihood that statements encountered during the exposure phase are true. In the experiments in which accuracy-focus instructions eliminated the illusory-truth effect, the exposure-phase statements were equally likely to be true or false (Brashier et al., 2020; Calvillo & Smelter, 2020; Nadarevic & Erdfelder, 2014). Hence, within the context of these experiments, prior exposure was indicative of neither truth nor falsity. Participants may thus have disregarded cues of prior exposure in their test-phase truth judgments, adjusting their interpretation of the cues to their experienced validity. This line of thinking leads to the central hypothesis tested in Experiment 2: If participants focus on accuracy in an exposure phase in which the majority of the statements summarize scientific studies, they should be more likely to interpret cues of prior exposure as being indicative of truth; conversely, if participants focus on accuracy in an exposure phase dominated by advertising, they should be more likely to interpret cues of prior exposure as being indicative of falsity. These predicted effects of low versus high advertising exposure on the test-phase truth judgments should be observed for the repeated statements that were presented during the exposure phase, but not for new statements that were not encountered during the exposure phase. Unlike the truth-tagging hypothesis explicated in the previous paragraph, this contextual-association hypothesis does not rely on the retrieval of the individual response to a specific statement. Instead, the contextual-association hypothesis implies that the effect is driven by an association between repeated statements and the exposure phase, which participants perceive as being characterized by a high proportion of advertising or study statements. The contextual-association hypothesis thus implies an interaction between advertising exposure and the contrast between new and repeated statements on the test-phase truth ratings, reflecting that statements presented during high advertising exposure should receive decreased truth ratings compared to new statements.

### Methods

#### Participants

Participants were recruited via the online-access-panel provider Cint (https://www.cint.com/). Only individuals who had not participated in Experiment 1 were invited to participate. We aimed at collecting valid data of about 500 participants and ended data collection the day this criterion was reached. The data of 76 participants who had started the exposure phase had to be excluded because these participants did not complete the experiment or withdrew their consent into the use of their data. The final dataset comprised the data of *N* = 519 participants (263 females, 250 males, and 6 diverse) with a mean age of 46 (*SD* = 15) years, most of whom (506) were German native speakers. The sample was characterized by a diverse range of education. Participants were randomly assigned to either the low-advertising-exposure group (*n* = 259) or the high-advertising-exposure group (*n* = 260). A sensitivity analysis with G*Power (Faul et al., 2007) showed that, with a sample size of *N* = 519 participants and a multivariate approach to repeated-measures analyses, an interaction between statement type and advertising exposure on the truth ratings as small as η_p_^2^ = 0.03 could be detected at an α level of 0.05 with a statistical power of 1–β = 0.95.

#### Materials and Procedure

Materials and procedure were identical to those of Experiment 1, except that participants were asked to judge the veracity of the product statements on a scale ranging from “definitely false” to “definitely true” in the exposure phase.

### Results

#### Exposure-phase truth ratings

**Fig. 3:**
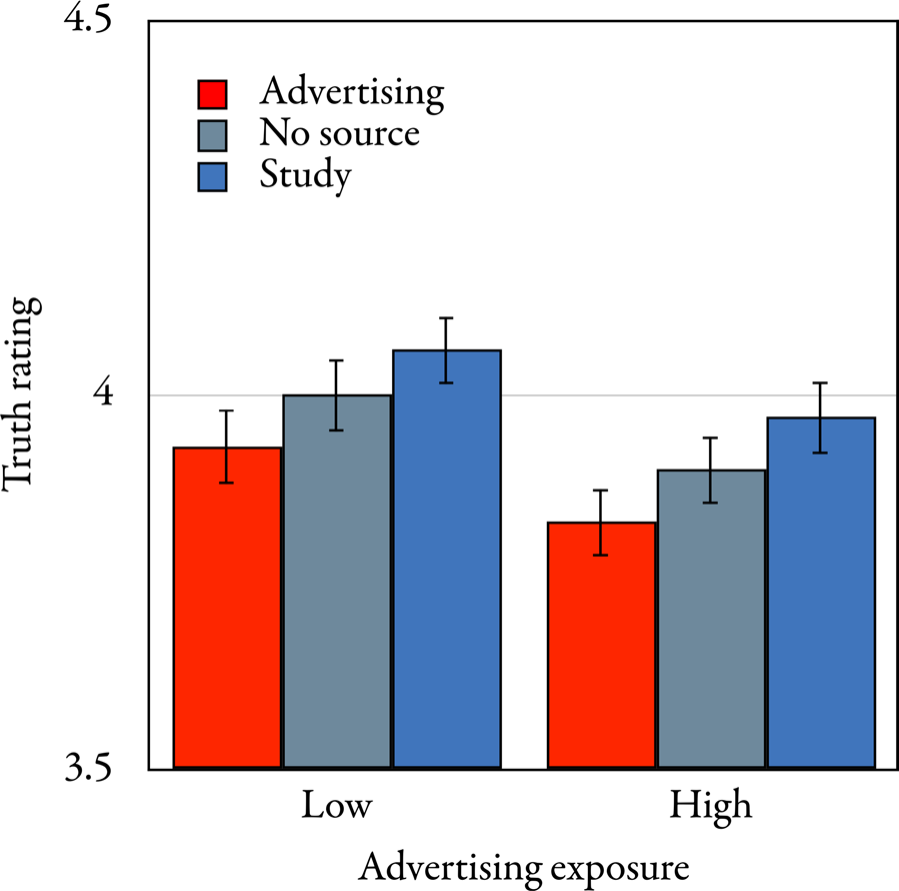
Mean exposure-phase truth ratings on a scale ranging from 1 (*definitely false*) to 6 *(definitely true*) as a function of (advertising, no source, or study) source type and (low or high) advertising exposure in the exposure phase of Experiment 2. The error bars represent the standard errors of the means

The mean exposure-phase truth ratings are displayed in Fig. 3. As in Experiment 1, a multivariate approach to repeated-measures analysis was used, with all multivariate test criteria corresponding to the same exact *F*-statistic which is reported. A 3 × 2 repeated-measures analysis of variance with statement type (advertising, no source, and study) as a repeated-measures factor, advertising exposure (high and low) as a group factor and exposure-phase truth ratings as the dependent variable revealed that statement type had a significant effect on the exposure-phase truth ratings, *F*(2,516) = 20.49, *p* < 0.001, η_p_^2 ^= 0.07. Helmert contrasts confirmed that the advertising source was associated with lower exposure-phase truth ratings than the other sources, *F*(1,517) = 35.52, *p* < 0.001, η_p_^2^ = 0.06, and that statements for which no source was displayed were associated with lower exposure-phase truth ratings than study statements, *F*(1,517) = 13.56, *p* < 0.001, η_p_^2^ = 0.03.

Advertising exposure had no significant main effect on the exposure-phase truth ratings, *F*(1,517) = 2.23, *p* = 0.136, η_p_^2 ^< 0.01. Advertising exposure did not significantly interact with statement type, *F*(2,516) = 0.09, *p* = 0.918, η_p_^2 ^< 0.01.

#### Test-phase truth ratings

**Fig. 4:**
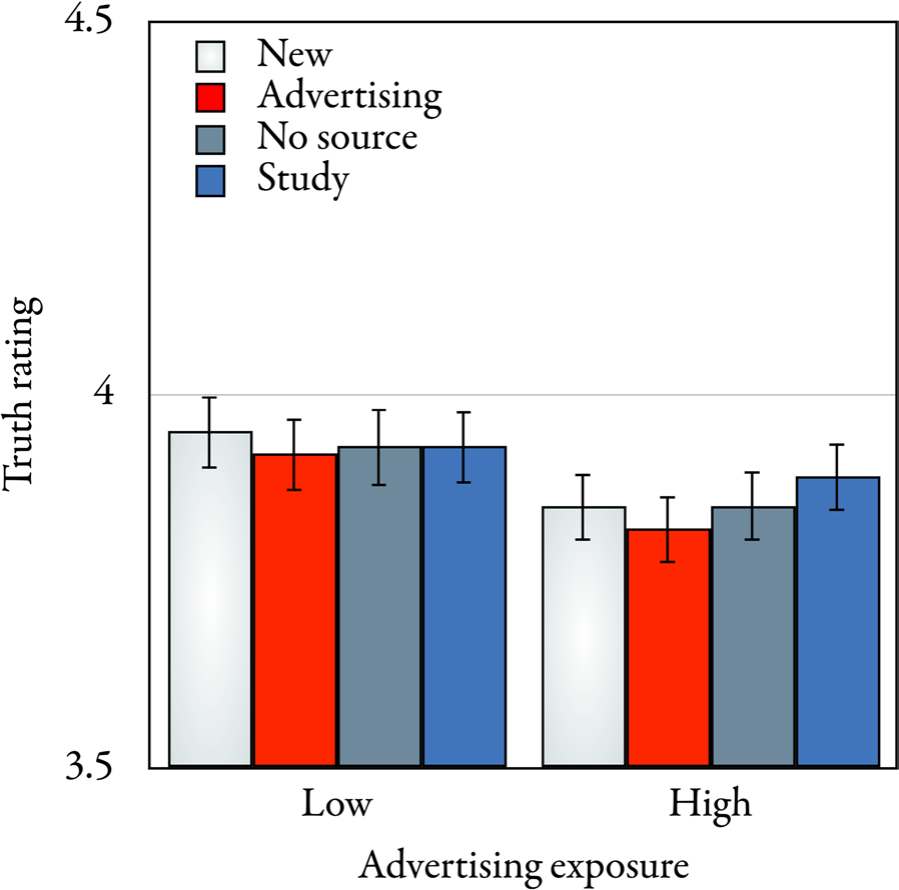
Mean test-phase truth ratings on a scale ranging from 1 (*definitely false*) to 6 *(definitely true*) as a function of (new, advertising, no source, or study) statement type and (low or high) advertising exposure in the test phase of Experiment 2. The error bars represent the standard errors of the means

The mean test-phase truth ratings are displayed in Fig. 4. A 4 × 2 repeated-measures analysis of variance with statement type (new, advertising, no source, and study) as a repeated-measures factor, advertising exposure (high and low) as a group factor and test-phase truth ratings as dependent variable revealed that statement type had a significant effect on the test-phase truth ratings, *F*(3,515) = 2.92, *p* = 0.034, η_p_^2^ = 0.02. Helmert contrasts were used to analyze the differences in test-phase truth ratings across the statement types. Test-phase truth ratings did not significantly differ between new and repeated statements, *F*(1,517) = 1.85, *p* = 0.175, η_p_^2^ < 0.01, which shows that the illusory-truth effect was absent in Experiment 2. Advertising statements led to lower truth ratings than the other repeated statements, *F*(1,517) = 4.57, *p* = 0.033, η_p_^2^ = 0.01. Truth ratings did not significantly differ between statements for which no source was displayed and study statements, *F*(1,517) = 2.19, *p* = 0.139, η_p_^2 ^< 0.01. In summary, no illusory-truth effect was obtained but the advertising source had a significant effect on the test-phase truth ratings in Experiment 2.

Advertising exposure had no significant main effect on the test-phase truth ratings, *F*(1,517) = 1.57, *p* = 0.211, η_p_^2 ^< 0.01. Advertising exposure did not significantly interact with statement type, *F*(3,515) = 2.42, *p* = 0.065, η_p_^2 ^= 0.01, and it also did not significantly interact with the contrast between new and repeated statements, *F*(1,517) = 2.47, *p* = 0.117, η_p_^2 ^< 0.01.

### Discussion

In line with previous studies (Brashier et al., 2020; Calvillo & Smelter, 2020; Nadarevic & Erdfelder, 2014), the illusory-truth effect was absent when the exposure-phase instructions required participants to process the product statements with a focus on accuracy. Other than in Experiment 1, the test-phase truth ratings did not differ significantly between repeated and new statements despite the large sample size that ensured a high statistical sensitivity to detect an illusory-truth effect if it existed (given a sample size of *N* = 519 participants, an illusory-truth effect—defined as the contrast between the new statements and all other types of statements—as small as η_p_^2^ = 0.02 could be detected at an α level of 0.05 with a statistical power of 1 – β = 0.95). Furthermore, there was no significant interaction between statement type and advertising exposure, indicating that the illusory-truth effect was absent regardless of the proportion of information from advertising or scientific studies encountered in the exposure phase of Experiment 2. The interaction between advertising exposure and the contrast between new and repeated statements was not significant, despite the large sample size, which provided considerable sensitivity to detect an effect if it existed, and the associated sample effect size was negligible (η_p_^2^ < 0.01). These findings provide evidence against the contextual-association hypothesis, according to which the absence of the illusory-truth effect under accuracy-focused processing depends on a contextual episodic representation of the proportion of statements from trustworthy and untrustworthy sources encountered in the exposure phase. Instead, the results suggest that the absence of the illusory-truth effect under accuracy-focused processing may result from factors other than the proportion of trustworthy and untrustworthy sources encountered during the exposure phase.

The pattern of results aligns more closely with the truth-tagging hypothesis. Unlike in Experiment 1, the source associated with the specific statements in the exposure phase continued to affect the test-phase truth ratings, as advertising statements received truth ratings that were slightly but significantly lower than those of other types of repeated statements. This effect was independent of the proportion of these sources among the exposure-phase statements. Together, this suggests that participants may occasionally succeed in retrieving “true” or “false” tags stored with the statements during the truth evaluations in the exposure phase (Nadarevic & Bell, 2024). Possibly, people refrain from heuristically inferring the truth or falsity of statements based on processing fluency when references to previous truth evaluations can occasionally be retrieved from memory. However, it is important to note that the effect of the advertising source on test-phase truth ratings was relatively small (η_p_^2^ = 0.01). This finding aligns with the general understanding that source memory is fragile (Johnson et al., 1993), which may also explain why a delay of the test phase by 1 week has been found to reinstate the illusory-truth effect even under accuracy-focused processing instructions (Nadarevic & Erdfelder, 2014).

## General discussion

Repeated statements often receive higher truth ratings than new statements. This illusory-truth effect has been proven to be an extremely robust phenomenon (Dechêne et al., 2010). In fact, arguments can be found that there may be some validity in trusting information that we have encountered before. For instance, we may surround ourselves with trustworthy people, and we will seek out newspapers and online sources that provide reliable information about the state of the world. Therefore, it seems reasonable to assume that in everyday contexts, there is an ecological correlation between the truth of a statement and the increased processing fluency that comes with repetition (Brashier & Marsh, 2020; Reber & Unkelbach, 2010). All else being equal, a statement may be more likely to be true if we have encountered it before than if we have never encountered it before. Therefore, it may be adaptive to rely on processing fluency for making judgments of truth. However, advertising sources may exploit this heuristic by repeating misleading advertising statements until consumers start to believe them, thereby transforming repetition into a tool for deception (Skurnik et al., 2005). Hence, the validity of cues of prior exposure as reliable indicators of truth may be called into question in contexts in which people are exposed to untrustworthy sources. Unkelbach and Stahl (2009) showed the interpretation of processing fluency to change when participants were instructed that *all statements in the exposure phase were false*. These findings suggest that there is some flexibility in how processing fluency is heuristically used when encountering repeated and new statements (see also Corneille et al., 2020; Scholl et al., 2014; Skurnik et al., 2000; Unkelbach, 2007). However, everyday situations are more ambiguous than experimental conditions in which all statements are false. For instance, people may be frequently exposed to varying levels of advertising that does not necessarily have to contain blatantly false information but is still a biased source that cannot be trusted to provide impartial information (Boush et al., 2009; Friestad & Wright, 1994). The purpose of the present experiments was to examine whether the illusory-truth effect is affected by such varying levels of advertising exposure.

In Experiment 1, participants were asked to read statements about products in the exposure phase and to rate the truth of repeated and new statements in the test phase. In line with the previous findings (Dechêne et al., 2010), repeated statements received higher truth ratings than new statements. This illusory-truth effect was independent of the source of the statements and the relative proportion of advertising or scientific sources. This insensitivity to the context of high or low advertising exposure demonstrates that people do not necessarily adjust their heuristic assessments of truth to the challenges posed by their environments. Specifically, they may be reluctant to abandon a heuristic that can be considered to be generally useful in most environments (Brashier & Marsh, 2020) even though this may make them vulnerable to manipulation by repeated messages from biased sources (e.g., Pennycook et al., 2018). These findings align well with the truth-by-default model (Levine, 2014), which suggests that people tend to accept information as true by default, irrespective of the context, unless specific triggers prompt a deliberate evaluation of its truthfulness.

In Experiment 2, participants evaluated the truth of the statements both in the exposure phase and in the test phase. Unlike in Experiment 1, the test-phase truth ratings did not significantly differ between the repeated and the new statements. This is in line with the previous studies demonstrating that requiring participants to focus on accuracy when processing the statements in the exposure phase eliminates the illusory-truth effect (Brashier et al., 2020; Calvillo & Smelter, 2020; Nadarevic & Erdfelder, 2014). However, just as in Experiment 1, there was no significant interaction between statement type and advertising exposure despite a large sample size that ensured a high sensitivity to detect such an interaction if it existed. The relative proportion of statements from advertising or scientific sources thus did not significantly affect how the truth of repeated statements was judged relative to that of new statements. Together, the findings of both experiments suggest that the relative proportion of information from trustworthy and untrustworthy sources has no appreciable impact on the illusory-truth effect.

When interpreting these findings, it is important to keep in mind that we deliberately implemented a less-than-extreme manipulation of contextual cues of truthfulness. Specifically, rather than instructing participants that *all* or *none* of the information provided in the exposure phase was false (cf. Unkelbach & Stahl, 2009), we varied the proportion of information from advertising and scientific studies. While information from advertising is not always blatantly false and information from scientific studies is not always perfectly true, the sources can be expected to differ in their trustworthiness, with advertising being obviously biased and scientific studies expected to be impartial. With 60% of the statements originating from advertising and only 20% statements from scientific studies, the high-advertising condition represents a high-advertising environment. This scenario is still realistic for websites that feature a mix of advertising and editorial content, thus reflecting the actual conditions under which consumers may encounter information in their daily lives. The present experimental manipulation thus models the everyday challenge of judging the truth in situations in which consumers are confronted with a varying mix of sources of information, some of which are trustworthy and some of which are not, making it difficult to clearly deduce the veracity of information (Boush et al., 2009; Friestad & Wright, 1994).

A limitation of the present experiments is that, because the manipulation of advertising exposure did not significantly affect test-phase truth ratings, the results do not directly demonstrate that participants processed the difference between high- and low-advertising-exposure contexts. However, an advantage of the present procedure is that it closely followed that of a previous study (Bell et al., 2022) in which highly similar manipulations of advertising exposure had significant effects on source monitoring and source guessing when participants were directly asked whether the test-phase statements corresponded to advertising statements, study statements, statements without a source, or new statements. Specifically, there was evidence that participants strongly relied on the relative proportion of advertising and study statements in the exposure phase for inferring the source of a test-phase statement if source memory was absent. Together, these findings suggest that the relative proportions of advertising and study statements during the exposure phase are processed, represented and remembered if directly probed, but people do not seem to spontaneously rely on this representation when making truth judgments.

In summary, the results of Experiment 1 show that the illusory-truth effect can be robustly obtained even in a condition in which people are exposed to high levels of advertising. Furthermore, the results of Experiment 2 corroborate previous findings showing that exposure-phase instructions prompting participants to focus on accuracy eliminate the illusory-truth effect (Nadarevic & Erdfelder, 2014). Just as the presence of the illusory-truth effect in Experiment 1, the absence of the illusory-truth effect in Experiment 2 was found to be independent of the level of advertising exposure. These findings demonstrate that the relative proportion of information from trustworthy and untrustworthy sources, representative of realistic contexts, does not significantly influence the presence or absence of the illusory-truth effect, indicating that the strategies that people adopt to judge the truth of information are robust across conditions of high and low advertising exposure.

## Data Availability

The data of the experiments and sensitivity analyses are available at: https://osf.io/nd85j/.
